# MCC950 Ameliorates Acute Liver Injury Through Modulating Macrophage Polarization and Myeloid-Derived Suppressor Cells Function

**DOI:** 10.3389/fmed.2021.752223

**Published:** 2021-11-19

**Authors:** Wei Yan, Yingchun Shen, Jinny Huang, Ling Lu, Qian Zhang

**Affiliations:** ^1^Hepatobiliary Center, The First Affiliated Hospital, Nanjing Medical University, Nanjing, China; ^2^Department of Surgery, School of Medicine, Johns Hopkins University, Baltimore, MD, United States; ^3^Division of Allergy and Clinical Immunology, School of Medicine, Johns Hopkins University, Baltimore, MD, United States; ^4^Department of General Surgery, The First Affiliated Hospital of Nanjing Medical University, Nanjing, China

**Keywords:** MCC950, acute liver injury, macrophage polarization, myeloid-derived suppressor cells, treatment

## Abstract

Acute liver injury (ALI) raises high mortality rates due to a rapid pathological process. MCC950, a highly selective nod-like receptor family pyrin domain containing 3 (NLRP3) inhibitor, has already been reported to show strong hepatoprotective effects in many different liver diseases. In this study, we unveiled the role of MCC950 in carbon tetrachloride (CCl_4_)-induced ALI and its underlying molecular mechanisms on days 1, 2, and 3. MCC950 could significantly inhibit liver injury, evidenced by decreased serum alamine aminotransferase (ALT) and aspartate aminotransferase (AST) levels on days 1 and 2, increased Albumin (ALB) level on day 3, and decreased histological score during the whole period. Moreover, lower M1 macrophage related to pro-inflammatory genes expression was observed in MCC950-treated ALI mice on day 1, while MCC950 pretreatment also polarized macrophage to M2 phenotype indicating anti-inflammatory response on days 2 and 3. Additionally, MDSC was significantly increased in blood, liver, and spleen in ALI mice at different time courses. Specifically, upregulated myeloid-derived suppressor cell (MDSC) proportions were found in blood and spleen on days 1 and 2, but showed decreased trend on day 3. However, liver MDSC numbers were increased on days 2 and 3, but no significance on day 1. In conclusion, MCC950 pretreatment alleviates CCl_4_-induced ALI through enhanced M2 macrophage and MDSC function at different time points of ALI. Further understanding of MCC950 in ALI may be a new potential therapeutic strategy.

## Introduction

Acute liver injury (ALI) has a rapid pathological process and is associated with a high mortality rate. It is already well-known that liver injury can be triggered by toxic chemicals, viruses, autoimmune diseases, and other factors, but there are currently no effective treatments (1). Therefore, it is necessary to investigate novel methods and drugs that can be used to treat the damage caused by acute liver injury. Carbon tetrachloride (CCl_4_), oxidized by cytochrome P450 2E1 (CYP2E1) to generate highly reactive free radical trichloromethyl radical (·CCl_3_) and trichloromethyl peroxy radical (·OOCCl_3_) in the liver, has been widely used to construct the liver injury models both *in vivo* and *in vitro* (2, 3).

The pathogenesis mechanism for ALI contains a series of complicate processes such as inflammation, oxidative stress, and autophagy (4, 5). Among them, inflammation is the most common trigger for ALI (6). Among many known inflammatory cell complexes, the nod-like receptor (NLR) family pyrin domain containing 3 (NLRP3) inflammasome activation, which is composed of NLRP3, adaptor apoptosis-associated speck-like protein containing a caspase recruitment domain (ASC), caspase-1, interleukin-1β (IL-1β), and interleukin-18 (IL-18), is a well-characterized inflammatory factor in development of ALI (7–9). Hence, targeting on inhibiting NLRP3 inflammasome and investigating potential mechanism may be a crucial and effective aspect in liver injury. MCC950 is one of the most potent and selective NLRP3 inhibitors discovered to date and it can bind directly and specifically to NLRP3, irrespective of its activation state (10). More recently, MCC950 was reported to alleviate chronic cholestatic liver injury (11), fulminant hepatitis (12), and liver fibrosis (13). However, little is known about the role of MCC950 treatment in CCl_4_-induced acute liver injury.

The myeloid-derived suppressor cell (MDSC) population consists of a variety of heterogeneous immature myeloid cells and is a significant component of the immunosuppressive network (14). The therapeutic role of MDSCs in many different immune diseases such as liver failure and cancer has been explored due to their important role in immune suppression. Recently, it was discovered that in Acetaminophen (APAP)-induced liver failure, Tumor Necrosis Factor Alpha (TNF-α)/LipoPolySaccharide (LPS) MDSCs served a protective role by reducing intrahepatic infiltration of activated neutrophils (15). Additionally, in melanoma cells, NLRP3 activation can induce the expansion and immune evasion of MDSCs (16). Currently, there is no study on the role of MDSCs and MCC950 in ALI.

In liver diseases, the M2 macrophage participates in tissue repair and resolution of inflammation, whereas the M1 phenotype results in pro-inflammatory signaling based on their functions, secreted cytokines, and transcriptional profiles (17, 18). Moreover, inhibiting NLRP3-mediated M1 macrophage polarization in non-alcoholic steatohepatitis can lead to reduced liver steatosis and inflammation (19). However, the relationship between MCC950 and macrophage polarization in ALI still remains unknown.

In this study, we determined the effect of MCC950 treatment on CCl_4_-induced liver injury in a murine model. We first proved that MCC950 can alleviate CCl_4_-induced liver damage and we further provided evidence for the mechanism of protective effect of MCC950 against liver inflammation—MCC950 promotes M2 macrophage polarization and enhances MDSC function. All these data highlight the clinical potential of MCC950 as a treatment strategy for ALI.

## Materials and Methods

### Animals and Experimental Design

All the procedures involving mice were performed in accordance with the approved protocols from the Animal Care and Use Committee of the Johns Hopkins University School of Medicine. An 8-week-old male C57BL/6 mice were used to construct ALI mouse model by CCl_4_ (Sigma, 270652, MO, USA) dissolved in olive oil (1 mg/kg) *via* intraperitoneal injection. MCC950 (Cell Signaling Technology, 86428S, MA, USA) was dissolved in sterile water and injected (10 mg/kg) 1 h before CCl_4_ induction through intraperitoneal injection. Mouse was sacrificed and serum, blood, spleen, and liver tissues were collected for further detection on days 1, 2, and 3.

#### Histopathology and Immunofluorescence (IF)

The 4-μm liver paraffin sections were stained with H&E (Sigma, MO, USA) according to the instructions of the manufacturer and images were taken under light microscope (Nikon, Tokyo, Japan). Additionally, for IF staining, 4 μm liver frozen sections were fixed by paraformaldehyde for 10 min and washed with Tris-buffered saline with Tween (TBST) for three times and then blocked with 10% Fetal Bovine Serum (FBS) in TBST for 1 h at room temperature. The sections were then incubated with the primary antibodies against CD68 (1:100, BioRad, MCA1957, CA, USA) and Arg-1 (1:400, Proteintech, 16001-1, IL, USA) or isotype overnight at 4°C. The sections were washed with TBST and then incubated with fluorescent dye–conjugated secondary antibodies for 60 min at room temperature. The fluorescent-positive cells were evaluated by using ImageJ Software version 1.50e National Institutes of Health, USA. Four to six sections from each sample were used for analysis.

#### Ribonucleic Acid Extraction, Reverse Transcription, and Quantitative Real-Time PCR (RT-PCR)

Total RNA of liver tissues from different groups was extracted with the RNeasy Plus Mini Kit (QIAGEN, Hilden, Germany) according to the instructions of the manufacturer. Complementary DNA (cDNA) was synthesized through the High-Capacity RNA-to-cDNA Kit (Applied Biosystems, MA, USA). Quantitative RT-PCR was performed by the SYBR Green PCR Master Mix (Applied Biosystems, MA, USA) by using the 7300 Real-Time PCR System (Applied Biosystems, MA, USA). Quantitation of the relative expression levels of each gene was detected in triplicate and calculated by using the 2^−ΔΔ*C*T^ method. β-actin was used as an endogenous control. The primers used are provided in Table 1.

**Table 1:** Sequences of target genes for real-time PCR.

**Gene**	**Forward primer (5′ to 3′)**	**Reverse primer (5′ to 3′)**
β-actin (mouse)	GGTTGTCTCCTGCGACTTCA	TGGTCCAGGGTTTCTTACTCC
NLRP3 (mouse)	ATTACCCGCCCGAGAAAGG	TCGCAGCAAAGATCCACACAG
IL-1β (mouse)	GCAACTGTTCCTGAACTCAACT	ATCTTTTGGGGTCCGTCAACT
IL-6 (mouse)	TAGTCCTTCCTACCCCAATTTCC	TTGGTCCTTAGCCACTCCTTC
iNOS (mouse)	GTTCTCAGCCCAACAATACAAGA	GTGGACGGGTCGATGTCAC
Arg-1 (mouse)	CTCCAAGCCAAAGTCCTTAGAG	AGGAGCTGTCATTAGGGACATC
Ym1/2 (mouse)	CAGGTCTGGCAATTCTTCTGAA	GTCTTGCTCATGTGTGTAAGTGA
Fizz1 (mouse)	CCAATCCAGCTAACTATCCCTCC	ACCCAGTAGCAGTCATCCCA

### Western Blotting (WB)

For WB analysis, an equal amount of total protein (20–50 μg) was loaded onto a 12% Tris-Glycine Gel in the NuPAGE 2-morpholino-ethanesulfonic acid (MES) sodium dodecyl sulfate (SDS) Running Buffer (Thermo Fisher Scientific, MA, USA) and then transferred by using the iBlot 2 NC Stack System (Thermo Fisher Scientific, MA, USA). The membranes were blocked in 5% non-fat milk in TBST for 1 h at room temperature and probed with primary antibodies (NLRP3, 1:1,000; Novus Biologicals, 12,446, CO, USA; IL-1β, 1:1,000; R&D Systems, AF-401, MN, USA) overnight at 4°C. Species appropriate secondary antibodies conjugated to IRDye 680RD were used according to the instructions of the manufacturer. Band intensities were quantified by the Image J Software and quantification on each band was normalized to glyceraldehyde 3-phosphate dehydrogenase (GAPDH).

### Preparation of the Cell Suspension

After mice were anesthetized, blood was collected *via* central vein, Ammonium-Chloride-Potassium (ACK) lysis buffer was used to lyse red blood cells (RBCs), and then wash with phosphate-buffered saline (PBS). During dissection, spleens were removed and collected in RPMI-1640 solution (11875093, Thermo Fisher Scientific, MA, USA) on the ice. Single cells were obtained after mashing the spleen through a 70-μm nylon cell strainer (VWR International, PA, USA) followed by 10 min treatment with 5 ml RBC lysis buffer (420302, BioLegend, CA, USA) at room temperature. After washing with RPMI-1640 solution, cells were resuspended in RPMI-1640 + 2% FBS. Livers were collected and digested in 1 mg/ml collagenase II for 30 min in 37°C followed by being mashed through a 70-μm strainer to obtain the cell suspension containing hepatocytes and non-parenchymal cells (NPCs). The hepatocytes were removed by centrifugation at a speed of 50 Relative Centrifugal Force (RCF) for 5 min and then the NPCs were collected from the supernatant above after centrifugation at 400 rcf for 5 min. After 10 min of RBC lysis buffer, NPCs were suspended in RPMI-1640 solution.

### Flow Cytometry

Single-cell suspensions (2 × 10^5^) from blood, spleen, and liver were blocked with antimouse CD16/32 (1:100, BioLegend, 101302, CA, USA) diluted in PBS for 20 min and then stained with fluorescently-labeled antibodies against surface markers of MDSC (CD11b and Gr-1, 1:200, BioLegend, 101212 and 108408, CA, USA) for 40 min at 4°C. fluorescence-activated single cell sorting (FACS) analysis was performed on the FACSCalibur Flow Cytometer (BD Biosciences, CA, USA) by using the FlowJo Software (CA, USA).

### Statistical Analysis

All the experimental data were analyzed by using the GraphPad Prism (CA, USA) and were presented as the means with error bars showing the SEM (mean ± SEM). Analysis of differences was performed by using the two-tailed Student's *t*-test or with the ANOVA. *P*-values <0.05 were considered as statistically significant.

## Results

### MCC950 Alleviates Acute Liver Injury

**Figure 1:**
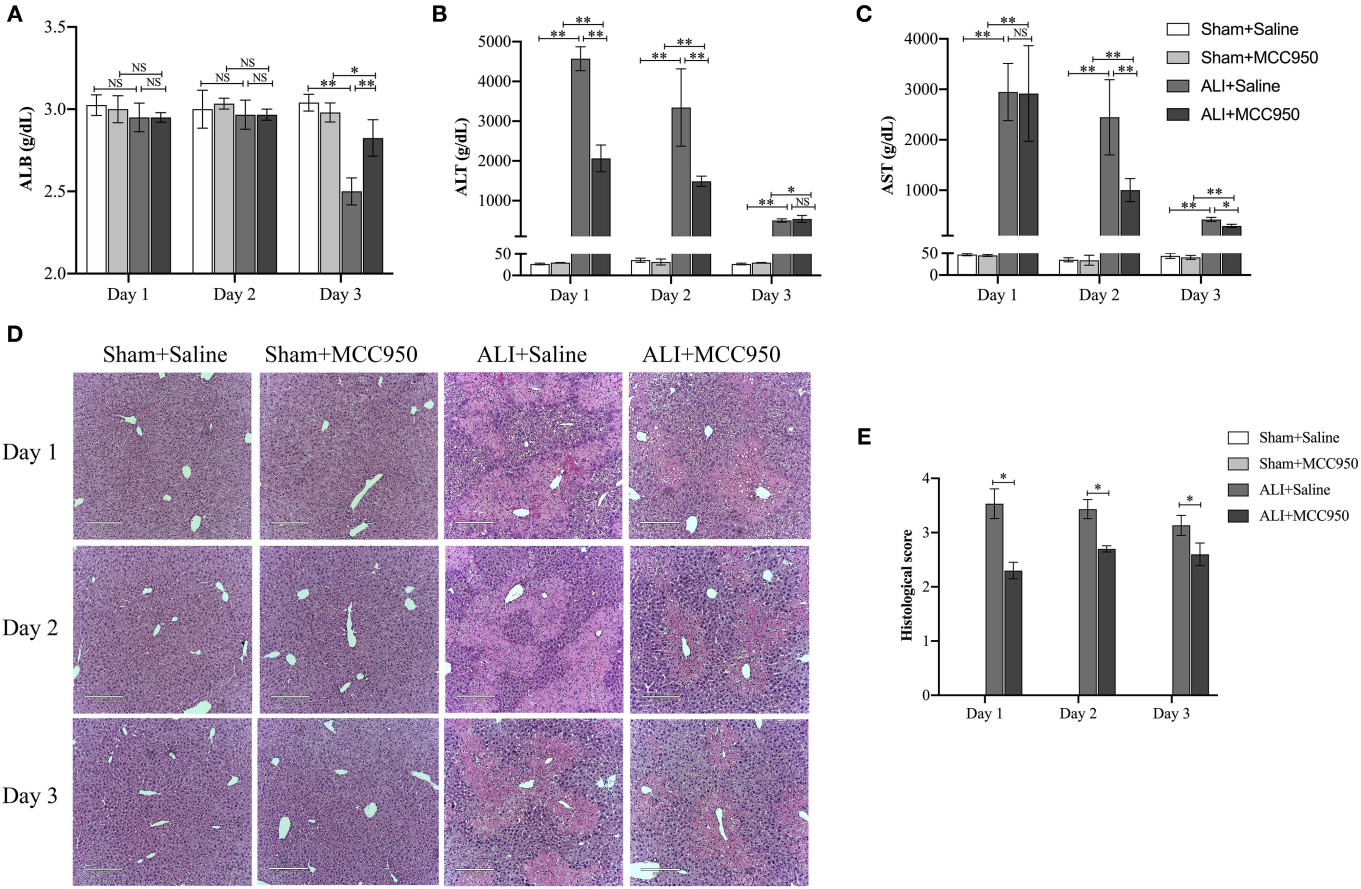
MCC950 alleviates acute liver injury. **(A)** Time course of ALB serum levels in different mice (*n* = 8). **(B)** Time course of ALT serum levels in different mice (*n* = 8). **(C)** Time course of AST serum levels in different mice (*n* = 8). **(D)** Histological examination of mouse paraffin liver sections stained with H&E staining from carbon tetrachloride (CCl_4_)-treated mice pretreated with vehicle or MCC950. **(E)** The histological scores for liver sections in different groups. Data are presented as mean ± SEM. NS: No significance. *p < 0.05, **p < 0.01. Intergroup differences are determined by the Student's *t*-test.

To better understand the role of NLRP3 inflammasome in ALI, MCC950, a highly selective NLRP3 inhibitor, was used to treat animals 1 h before CCl_4_ injection. The biochemical markers of hepatocellular damage, serum ALB (Figure 1A), ALT (Figure 1B), and AST (Figure 1C) concentration levels showed that CCl_4_ injection can lead to liver damage at different time points, while the most severe damage was observed on day 1. Interestingly, increased serum ALB level was observed on day 3, but no changes on days 1 and 2 (Figure 1A). Moreover, MCC950 treatment significantly reduced AST (Figure 1B) and ALT (Figure 1C) levels, especially on days 1 and 2, while no significant reduction occurred on day 3. Meanwhile, H&E staining showed that MCC950 treatment attenuated liver injury with less necrosis and inflammatory cell infiltration around the blood vessels at all the time points (Figures 1D,E). Given all the evidence, MCC950 indeed alleviates CCl_4_-induced ALI.

### MCC950 Inhibits Liver NLRP3 Inflammasome Activation in ALI Mice

**Figure 2:**
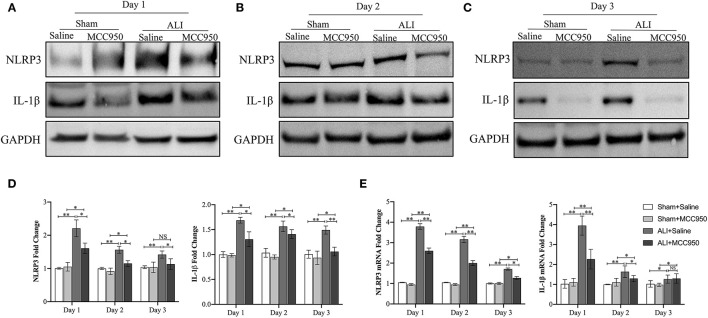
The nod-like receptor family pyrin domain containing 3 (NLRP3) inflammasome activation in acute liver injury mice is inhibited by MCC950. Western blot analysis of NLRP3 and interleukin-1β (IL-1β) protein level in liver tissues from CCl_4_-treated mice pretreated with vehicle or MCC950 on day 1 **(A)**, day 2 **(B)**, and day 3 **(C)**, GAPDH was detected as the loading control. **(D)** Quantitative analysis of western blots **(A–C)**, (*n* = 3). **(E)** Real-time PCR (RT-PCR) analysis of liver NLRP3 and IL-1β messenger RNA (mRNA) level in different mice. Data are presented as mean ± SEM. NS: No significance. *p < 0.05, **p < 0.01. Intergroup differences are determined by the Student's *t*-test.

As shown in Figure 2, the expression of NLRP3 and IL-1β in liver tissues was significantly increased in CCl_4_-induced ALI group compared with control group evaluated by WB (Figures 2A–D) and RT-PCR (Figure 2E) on days 1, 2, and 3. Moreover, MCC950 treatment markedly inhibited the expression of NLRP3 and IL-1β in ALI mice at different time points.

### MCC950 Ameliorates ALI *via* Enhanced MDSC Function

**Figure 3:**
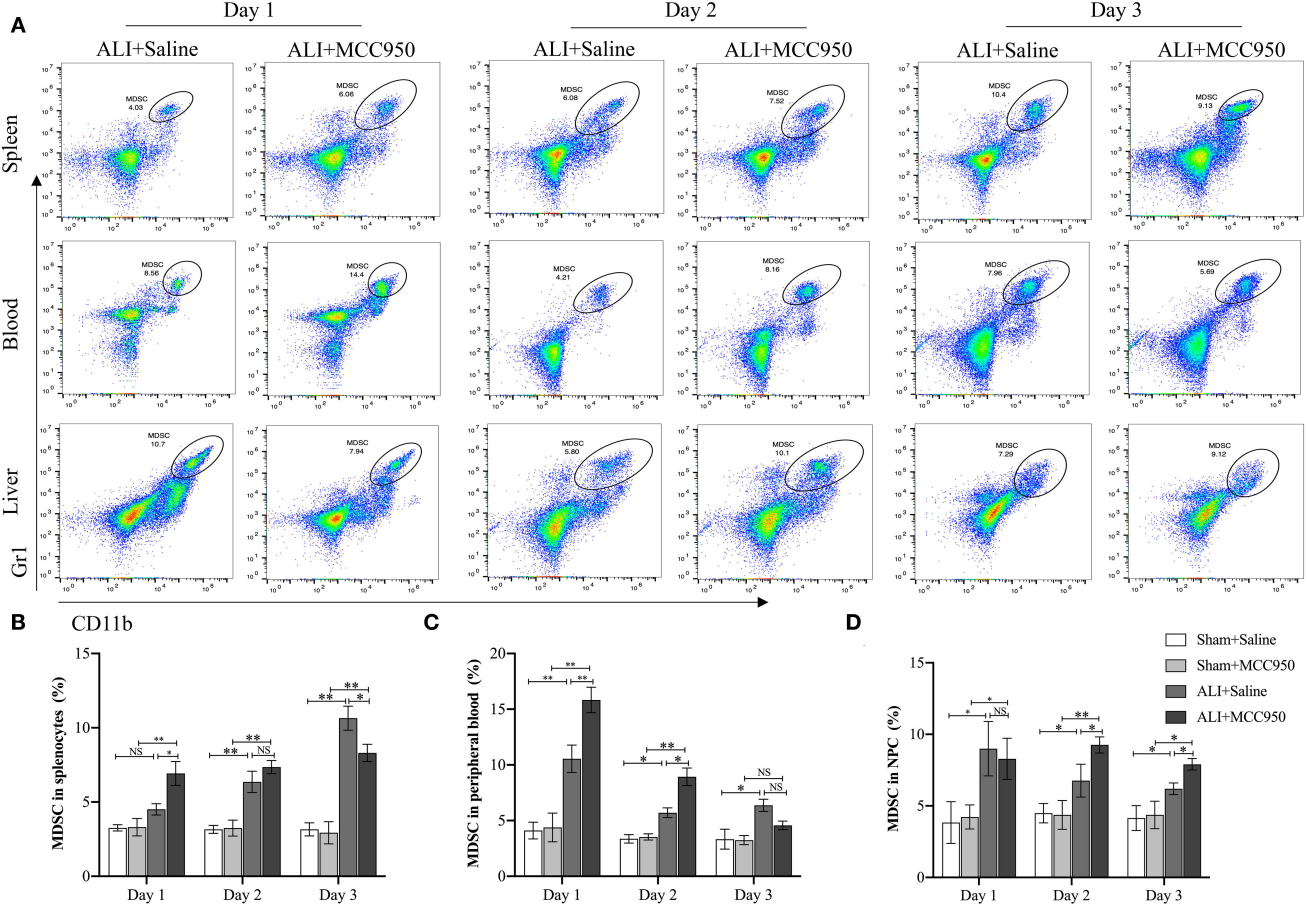
MCC950 treatment enhances myeloid-derived suppressor cell (MDSC) function in acute liver injury. **(A)** Representative images of MDSC in spleen, blood, and liver detected by flow cytometry in CCl_4_-treated mice pretreated with vehicle or MCC950 on days 1, 2, and 3. MDSC was marked as CD11b^+^Gr-1^+^. **(B)** Time points of MDSC percentages in spleen. **(C)** Time points of MDSC percentages in peripheral blood. **(D)** Time points of MDSC percentages in liver. Data are presented as mean ± SEM. NS: No significance. *p < 0.05, **p < 0.01. Intergroup differences are determined by the Student's *t*-test.

Next, we continued to use flow cytometry to assess the role of MCC950 treatment on MDSC function. As shown in Figure 3A and Supplementary Figure 1, MDSC numbers were increased in spleen, blood, and liver of ALI group compared with control group and sham group. Moreover, MCC950 treatment can upregulate spleen and blood MDSC proportions in days 1 and 2, but exist reduced tendency on day 3 (Figures 3B,C). However, liver MDSC numbers were increased on days 2 and 3, while no significance on day 1 (Figure 3D). Combine together, we proposed that MCC950 treatment can firstly increase spleen and blood MDSC on days 1 and 2 and then recruit MDSC into liver from days 2 to 3 during liver injury process.

### MCC950 Prevents ALI Through Polarizing Macrophage Into M2 Phenotype

**Figure 4:**
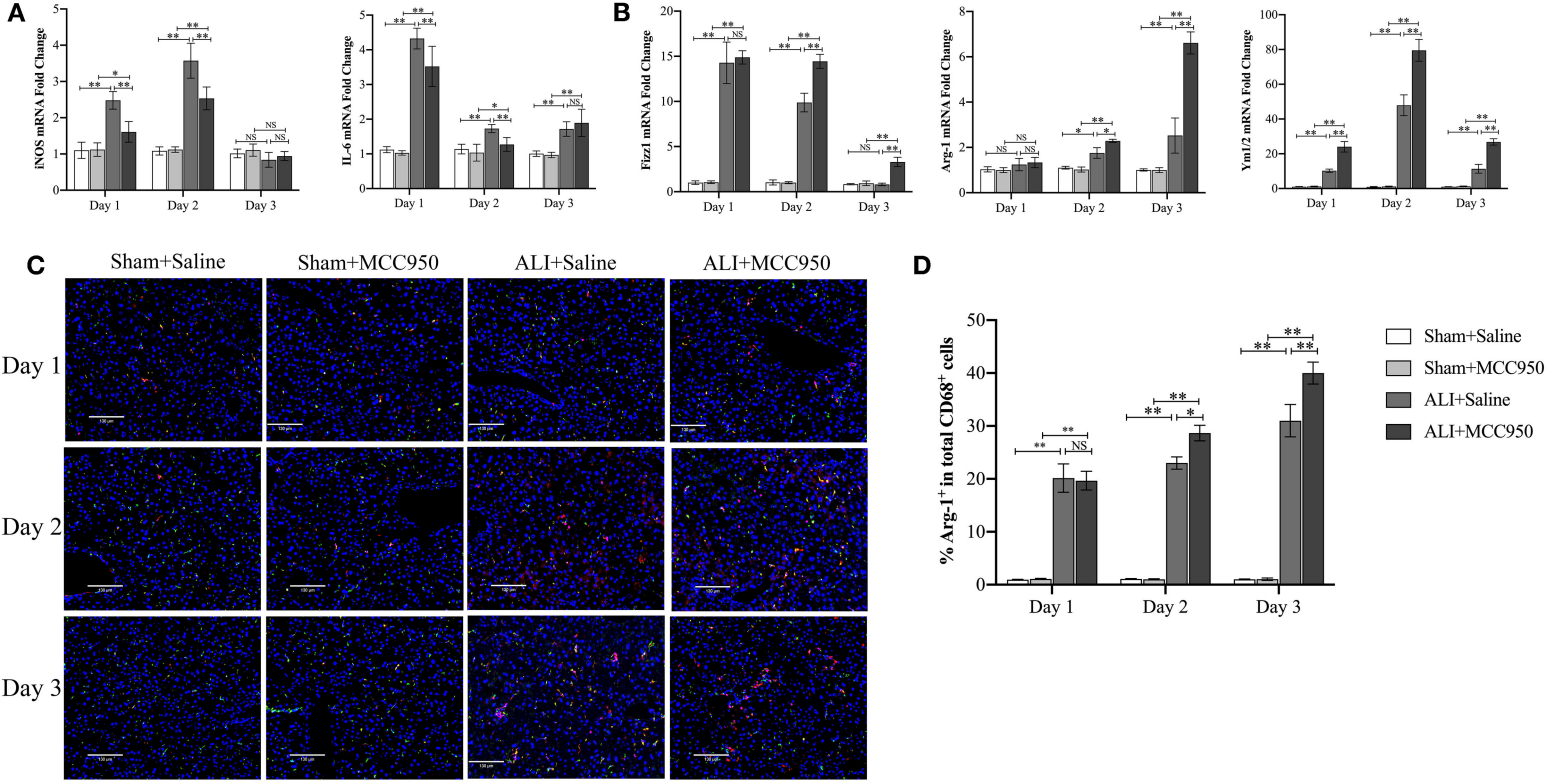
MCC950 prevents acute liver injury through polarizing macrophage into M2 phenotype. **(A)** Real-time PCR (RT-PCR) analysis of M1-related genes such as inducible nitric oxide synthase (iNOS) and interleukin-6 (IL-6) messenger RNA (mRNA) levels in liver tissues from CCl_4_-treated mice pretreated with vehicle or MCC950 on days 1, 2, and 3. **(B)** RT-PCR analysis of M2-related genes such as Fizz1, Arg-1, and Ym1/2 mRNA levels in liver tissues from CCl_4_-treated mice pretreated with vehicle or MCC950 on days 1, 2, and 3. **(C)** Representative images of double-immune fluorescence staining with CD68 and Arg-1 in frozen liver section from different mice. Nuclei were counterstained with 4′-6-diamidino-2-phenylindole dihydrochloride (DAPI). **(D)** Quantitation of liver tissue M2 macrophages as indicated by the percentage of CD68^+^Arg-1^+^ (%M2) among total CD68^+^ cells (*n* = 8–9 field/group). Data are presented as mean ± SEM. NS: No significance. ^*^p < 0.05, ^**^p < 0.01. Intergroup differences are determined by the Student's *t*-test.

To further investigate whether or not MCC950 attenuates liver damage through macrophage polarization, M1 [inducible nitric oxide synthase (iNOS) and interleukin-6 (IL-6)] and M2 (Fizz1, Arg-1, and Ym1/2) phenotypes were evaluated by RT-PCR and IF staining. As shown in Figure 4A, M1-related genes such as iNOS and IL-6 were reduced on days 1 and 2, but no significance on day 3. Additionally, all the M2-related genes were increased in ALI group and MCC950 treatment can lead to higher expression of Fizz1, Arg-1, and Ym1/2 on days 2 and 3, while no obvious significance on day 1 (Figure 4B). Moreover, we continued using IF costaining of CD68 and Arg-1 in the liver tissues. As shown in Figures 4C,D, double-positive cells were increased on days 2 and 3, while no changes on day 1, which are consistent with RT-PCR detection. Thus, these results prove that MCC950 attenuates ALI through polarizing macrophage into M2 phenotype on days 2 and 3.

### Cytokines Dysfunction Can Be Rescued by MCC950 Treatment in ALI

**Figure 5:**
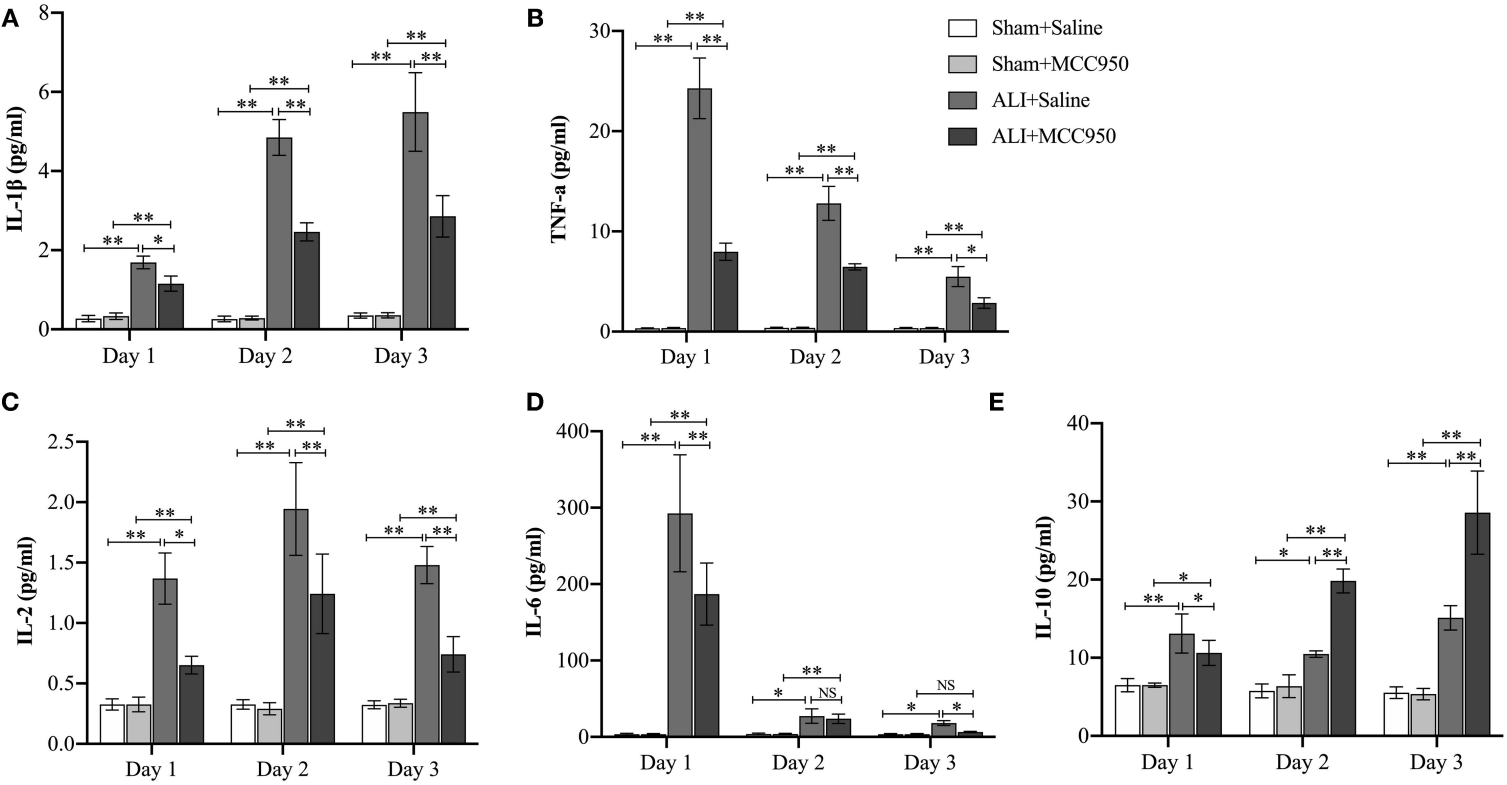
MCC950 treatment rescues cytokines dysfunction in acute liver injury. Levels of IL-1β **(A)**, TNF-α **(B)**, IL-2 **(C)**, IL-6 **(D)**, and IL-10 **(E)** in serum from mice in different groups. Data are presented as mean ± SEM. NS: No significance *p < 0.05, **p < 0.01. Intergroup differences are determined by the Student's *t*-test.

Finally, cytokines (IL-1β, IL-2, IL-6, IL-10, and TNF-α) in serum were detected in different time points (Figure 5). MCC950 treatment can reduce IL-1β, IL-6, and TNF-α (pg/ml) level on days 1, 2, and 3, while it can only reduce IL-6 (pg/ml) level on days 1 and 3. Of note, MCC950 also can enhance IL-10 (pg/ml) expression on days 2 and 3. These data indicate that MCC950 can rescue cytokines dysfunction in ALI.

## Discussion

Acute massive or chronic persistent liver damages can result in liver failure. Developing an alternative therapeutic stratagem to reduce injury, prevent progression, and restore liver function is of significant clinical relevance. In this study, we provided convincing evidence that pretreatment with MCC950, a NLRP3-specific inhibitor, effectively alleviates CCl_4_-induced ALI in a murine model.

Nowadays, inflammation is the most prevalent underlying pathology in ALI. It is well-documented that NLRP3 inflammasome plays a significant role in both the early and progressive inflammation (20, 21). Recently, several compounds have emerged as inhibitors for the NLRP3 inflammasome cascade (22); among all the inhibitors of NLRP3 inflammasome, MCC950 shows excellent potency and high target selectivity, yet its pharmacokinetic and toxicokinetic properties limited its therapeutic development in the clinical settings (10). Previous studies have demonstrated that MCC950 treatment could reduce IL-1β production and attenuate the severity of lung ischemia-reperfusion injury (23), ulcerative colitis (24), myocardial infarction (25), multiple sclerosis (22), and liver transplantation (26). Importantly, MCC950 exerts strong hepatoprotective properties in multiple types of mouse liver injury models. A recent study suggested that MCC950 exerts protective effects for liver inflammation and fibrosis in two models of Non-alcoholic steatohepatitis (NASH) (27). Also, in a bile duct ligation (BDL) model for cholestasis, MCC950 has been demonstrated to reduce liver fibrosis through inhibiting NLRP3 and the mechanism was partially attributed to inhibition of Toll-like receptor signaling (11). Additionally, MCC950 had also been reported to reduce liver inflammatory response and fibrosis of testosterone-treated mice (13). However, its efficacy in ALI remains unknown.

In this study, CCl_4_-induced ALI model was constructed with MCC950 or vehicle pretreatment. Mice were sacrificed during both the early phase (days 1 and 2) and also the late phase (day 3) in order to determine the mechanism of the treatment. Through detection of H&E staining, serum ALB, AST and ALT levels, and NLRP3 inflammasome levels, we found that activated NLRP3 and IL-1β expressions are coincident with the severity of histopathological damage in the liver. Moreover, MCC950 treatment actually blocked NLRP3 and IL-1β expression at different time points. Interestingly, MCC950 treatment in ALI mice can reduce liver injury and function at all the different time points, especially in the early phase days 1 and 2, indicating MCC950 can be viewed as alternative therapeutic target in ALI.

Recently, MDSCs have been gaining increased attention due to its ability to reduce inflammation and limit tissue damage by modulating both the innate and adaptive immune responses (28, 29). In this study, we found that for ALI mice, the MDSC population increased in spleen, blood, and liver tissues in both the early phase and the late phase after CCL_4_ injection. To investigate how MCC950 treatment affected MDSC population, we also evaluated the MDSC numbers in MCC950-treated mice at different time points. Notably, in the early phase, MCC950 treatment can increase MDSC numbers in spleen and blood, but not increase MDSC numbers in liver on day 1. Surprisingly, in the late phase (day 3), MCC950 can enhance MDSC number in liver, but reduced tendency in spleen and blood was observed. Accordingly, it is well-founded that enhanced MDSC numbers generated after MCC950 treatment can participate in rescuing process in the early phase and regeneration process in the late phase. However, the molecular mechanism through which driving MDSC mobilization into inflamed liver remains elusive. Upon NLRP3 activation, the inactive IL-1β precursor is processed by caspase-1 to active, mature IL-1β, which could induce cytokines associated with MDSC expansion such as IL-6 and IL-8 (30). A recent study suggested that IL-25 is highly expressed in both the human and mouse liver and plays a critical function in maintaining the homeostasis and limiting local inflammation through recruiting MDSC (31). Another study further demonstrated that during the pathogenesis of *Propionibacterium acnes*/LPS-induced fulminant hepatitis (FH), a protein kinase Tpl2 could mediate the induction of MDSC-attracting chemokines such as CXC chemokine ligand-1 (CXCL1) and CXC chemokine ligand-2 (CXCL2) through modulating IL-25 signaling in hepatocytes, which could further promote the recruitment of MDSC into liver (32). Moreover, CCL17 was also reported to be a MDSC-attracting chemokine induced by IL-25 in D-galactose (D-Gal)/LPS-induced fulminant hepatitis (FH) mice (31). These results support the function of MDSCs in tissue protection in terms of inflammation and provide evidence that MCC950 could rescue liver damage *via* recruiting MDSC to liver.

Generally, M1 macrophages are thought to promote cytotoxic/pro-inflammatory factors such as IL-1β, TNF-α, IL-6, and iNOS, which can cause cell apoptosis and tissue damage, whereas M2 macrophages are associated with tissue repair/reparative fibrosis *via* upregulating Fizz1, Arg-1, Ym1/2, and IL-10 (33–35). In addition, these M1 and M2 macrophages can modulate hepatic lesions induced by hepatotoxicants (36). Therefore, we decided to evaluate the effect of MCC950 on macrophage polarization in ALI. Using RT-PCR, we found that MCC950 treatment can upregulate M2-related genes (Fizz1, Arg-1, Ym1/2, and IL-10), but decrease M1-related genes (IL-1β, TNF-α, IL-6, and iNOS). Double IF analyses by CD68 and Arg-1 further supported our analysis.

As mentioned above, MCC950 can influence cytokine levels. We measured the levels of IL-1β, IL-2, IL-6, IL-10, and TNF-α in serum. Consistent with pathological results, MCC950 can alleviate liver damage through reducing IL-1β, IL-2, IL-6, and TNF-α, but enhancing IL-10 production. In line with our data, many other studies reported that MCC950 could reduce the production of pro-inflammatory cytokines including IL-1β, IL-18, IL-1α, IFNγ, TNF-α, IL-6, IL-17, and chemokine Macrophage Inflammatory Protein 1-? (MIP-1α) and inhibit neutrophil infiltration and cell apoptosis (37–39), which may be one of the reasons why MCC950 plays a role in suppressing inflammation and improving liver function in ALI.

In conclusion, this study demonstrated that MCC950 treatment in CCl_4_-induced ALI can recruit MDSCs, promote M2 macrophage polarization, and modulate cytokine levels by decreasing pro-inflammatory and increasing anti-inflammatory cytokines. These protective effects happen both during the early phase (days 1 and 2) and the late phase (day 3) post-injury. Due to its ability to suppress inflammation and improve liver function, MCC950 treatment has important protective effects in the progression of ALI and may lead to new therapeutic strategies for ALI.

## Data Availability Statement

The original contributions presented in the study are included in the article/Supplementary Material, further inquiries can be directed to the corresponding author/s.

## Ethics Statement

All procedures involving mice were performed in accordance with the approved protocol from the Animal Care and Use Committee of the Johns Hopkins University School of Medicine (No. MO18M233).

## Author Contributions

QZ and WY conceived and designed the study and finalized the manuscript. JH conducted the experiments, analyzed data, and edited the manuscript. LL and YS mainly edited the manuscript. All the authors read and approved the final version of the manuscript.

## Funding

This study was supported by grants from the National Natural Science Foundation of China (81971495, 81571564, and 91442117), the Chinese Academy of Medical Sciences (CAMS) Innovation Fund for Medical Sciences (No. 2019-I2M-5-035), and the National Science Foundation of Jiangsu Province (BRA2017533, BK20191490, and BE2016766).

## Conflict of Interest

The authors declare that the research was conducted in the absence of any commercial or financial relationships that could be construed as a potential conflict of interest.

## Publisher's Note

All claims expressed in this article are solely those of the authors and do not necessarily represent those of their affiliated organizations, or those of the publisher, the editors and the reviewers. Any product that may be evaluated in this article, or claim that may be made by its manufacturer, is not guaranteed or endorsed by the publisher.

## Supplementary Material

Supplementary Figure 1 Representative images of MDSC in spleen, blood, and liver detected by flow cytometry in sham group pretreated with vehicle or MCC950 on days 1, 2, and 3. MDSC was marked as CD11b^+^Gr-1^+^.
